# 

**Published:** 1881-07

**Authors:** 


Meetings of Dental Societies in July.—The Pennsylvania State Dental Society, and the Pennsylvania State Dental Examination Board, 26th, at Chautauqua Lake; Southern Dental Association, and the N. C. State Dental Association, 26th, at Ashville; Alabama Dental Association, 19th, at Selma; Iowa State Dental

Society, 5th, at Oskaloosa; Nebraska State Dental Society, 23d, at Fremont; New Jersey State Dental Society, 20th, at Long Branch; Wisconsin State Dental Association, 20th, at Milwaukee.

### HYMENEAL.

> "Domestic happiness, thou only bliss
> Of Paradise that has survived the fall."

CRANE—ECKARD.—On June 2d, at Abington, Pa., by J. R. Eckard, D. D., Charles L. Crane, M. D., of Sumter County, S. C., to Anna M., daughter of the officiating clergyman.

GLEAVES—KEENE.—On the 9th of June, by Rev. Campbell Fair, D. D., Dr. Charles W. Gleaves, of Wytheville, Virginia, to Laura, daughter of the late N. B. Keene, of New Orleans.

*We take the privilege of deciding what we shall publish, but the author only is responsible for his production.*

We have facilities which enable us to make liberal discounts to our cash subscribers on text and many other books, also Webster's Unabridged Dictionary, the various publications of Frank Leslie and Harper, Scribner's Monthly, Lippincott's Magazine, North American Review, Popular Science Monthly, &c., &c., also many of the leading daily and weekly news and pictorial papers.

AN EXTRA
INDUCEMENT
*To Subscribers.*
See the Advertisement of the Johnson Revolving Book Case in the May issue.

This column will be sold for advertising space six months during the year at *special rates*.

Money is at OUR RISK ONLY when sent by Post Office Money Order, Registered Letter, or Check on Banks in New York City, made payable to INDEPENDENT PRACTITIONER, 20 Courtlandt street, N. Y. City.

